# Supplementary figures and images for: De novo protein fold families expand the designable ligand binding site space
Source: PLoS Comput Biol. 2021 Nov 22;17(11):e1009620. doi: 10.1371/journal.pcbi.1009620 (PMC8648124; doi:10.1371/journal.pcbi.1009620)

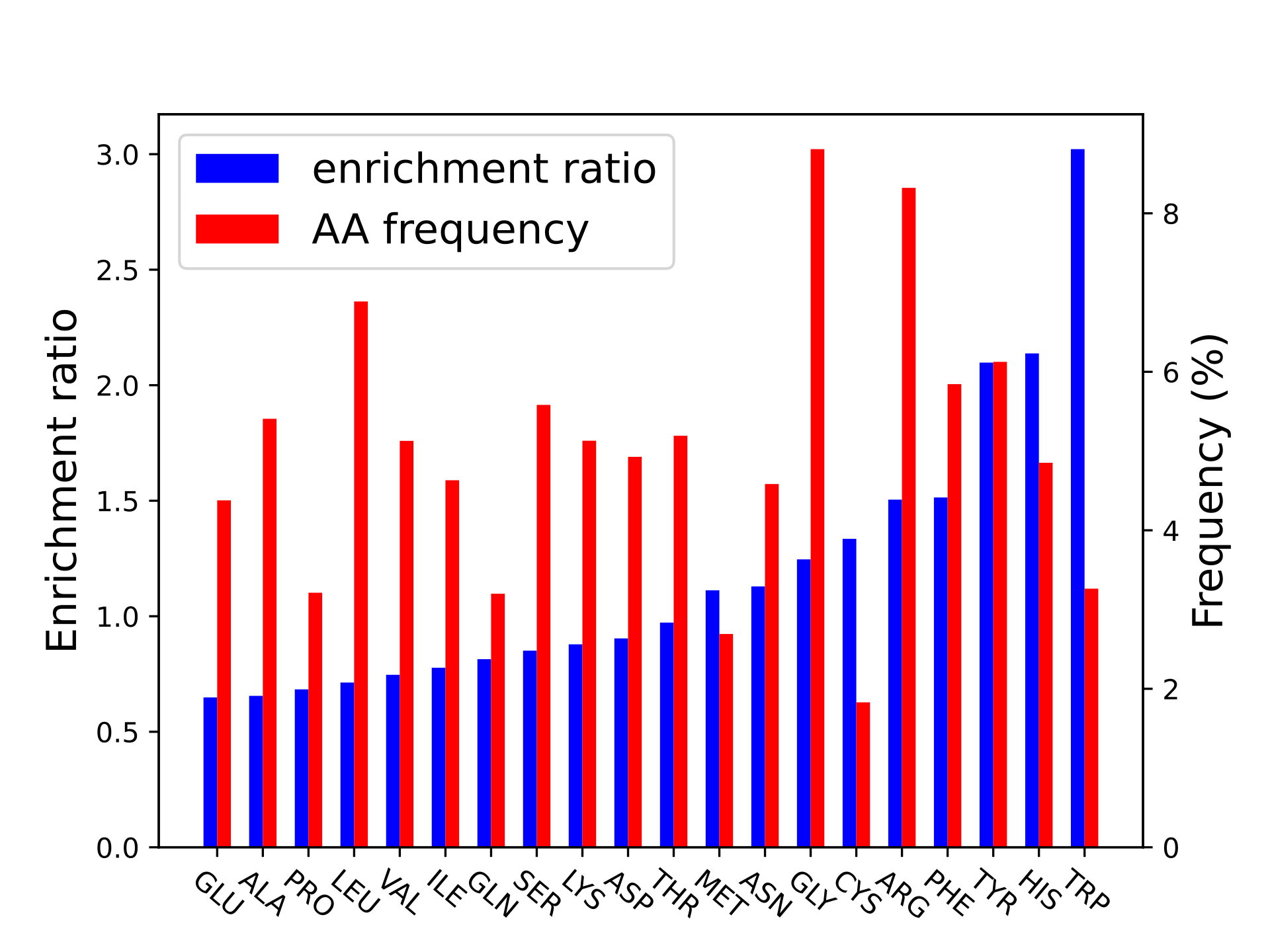

Supplement: S1 Fig — (TIFF) [file pcbi.1009620.s001.tiff]

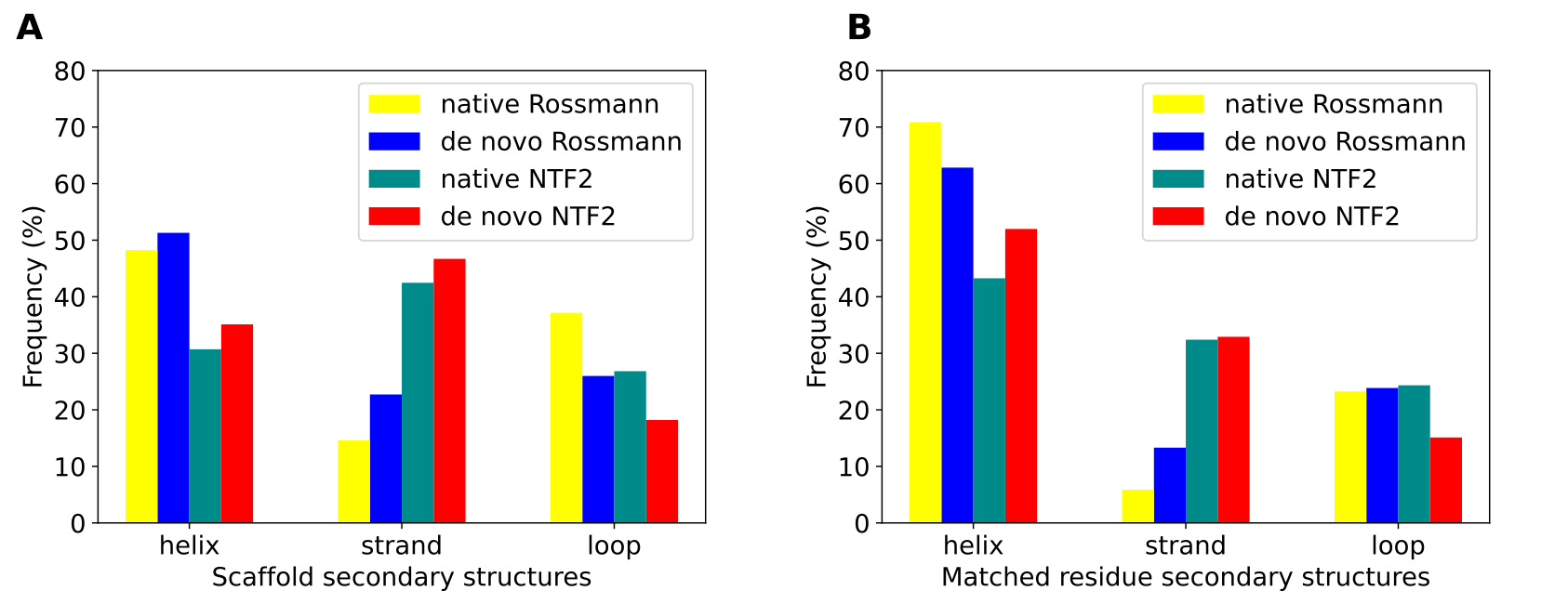

Supplement: S2 Fig — A. Distributions of secondary structures of scaffolds in different fold families. B. Distributions of secondary structures of matched 3-residue binding sites. (TIFF) [file pcbi.1009620.s002.tiff]

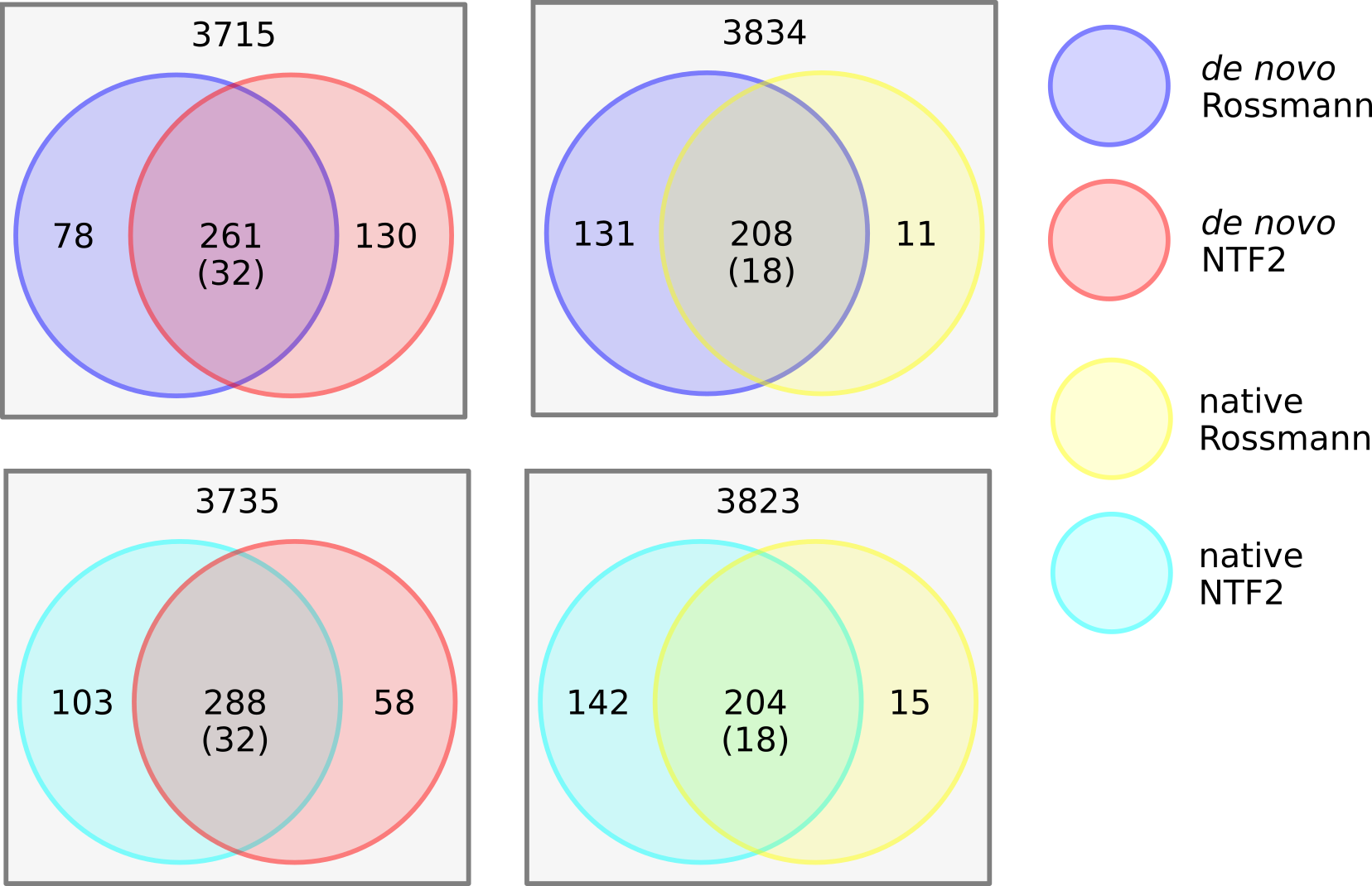

Supplement: S3 Fig — The 3-residue binding sites were clustered by 3D similarities of their ligands. A cluster is defined as matched if it has at least one matched binding site. For each Venn diagram, the number in the overlapping region is the observed number of clusters that can be matched to both scaffold sets, with the expected number in parentheses. The number in the non-overlapping region within a circle denotes the clusters that can only be matched to this scaffold set. The number outside the circles denotes the clusters that cannot be matched to either of the two scaffold sets. (TIFF) [file pcbi.1009620.s003.tiff]

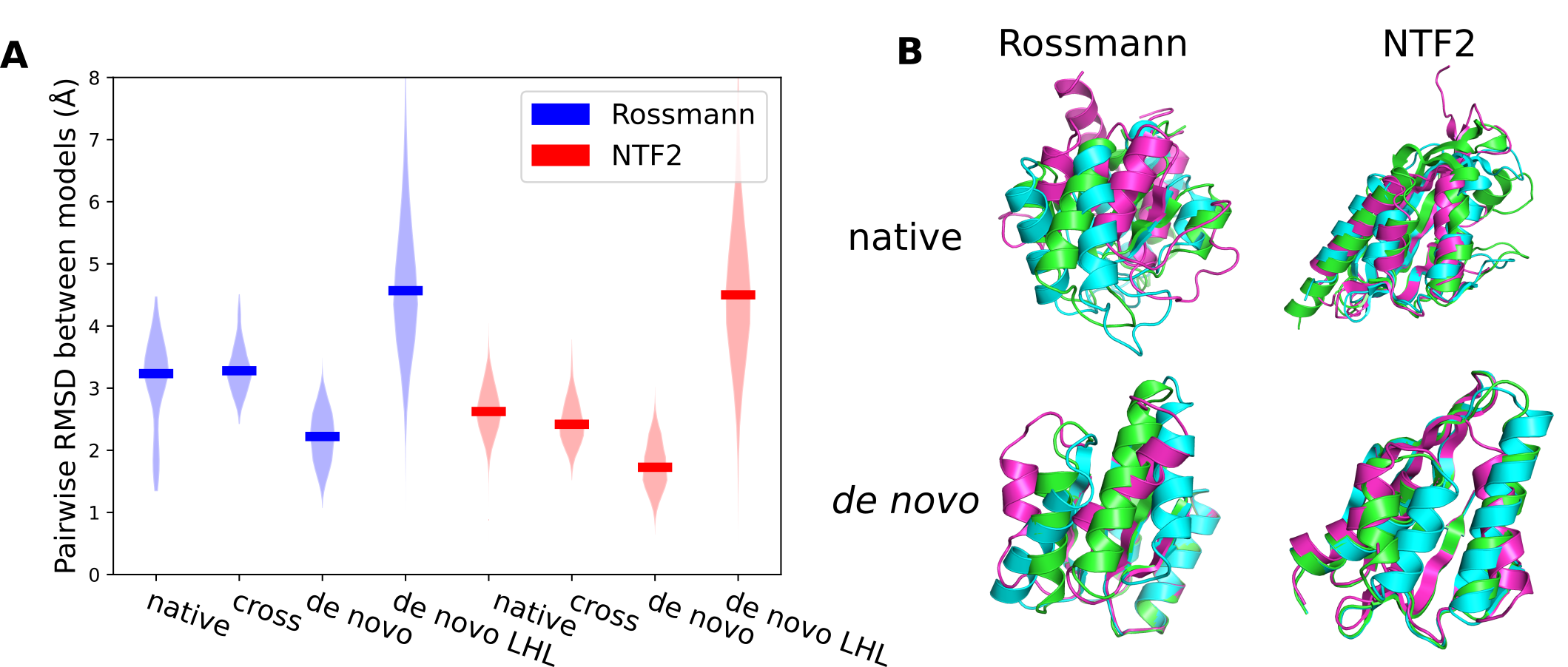

Supplement: S4 Fig — A. Distributions of pairwise RMSDs between structures in native fold families (native), de novo fold families (de novo), cross comparison between native and de novo fold families (cross), or pairwise RMSDs between the de novo designed LHL units when aligned the remainder of the protein (de novo LHL, calculated using the method in ref(16)). B. Examples of scaffold structures from each fold family. (TIFF) [file pcbi.1009620.s004.tiff]
